# AMANet: a data-augmented multi-scale temporal attention convolutional network for motor imagery classification

**DOI:** 10.3389/fnbot.2025.1704111

**Published:** 2026-01-09

**Authors:** Shu Wang, Raofen Wang, Liang Chang, Jianzhen Wu, Lingyan Hu

**Affiliations:** 1 School of Electronic and Electrical Engineering, Shanghai University of Engineering Science, Shanghai, China; 2 School of Mechatronic Engineering and Automation, Shanghai University, Shanghai, China

**Keywords:** attention mechanism, brain–computer interface, common spatial pattern, data augmentation, motor imagery

## Abstract

Motor imagery brain–computer interface (MI-BCI) has garnered considerable attention due to its potential for neural plasticity. However, the limited number of MI-EEG samples per subject and the susceptibility of features to noise and artifacts posed significant challenges for achieving high decoding performance. To address this problem, a Data-Augmented Multi-Scale Temporal Attention Convolutional Network (AMANet) was proposed. The network mainly consisted of four modules. First, the data augmentation module comprises three steps: sliding-window segmentation to increase sample size, Common Spatial Pattern (CSP) extraction of discriminative spatial features, and linear scaling to enhance network robustness. Then, multi-scale temporal convolution was incorporated to dynamically extract temporal and spatial features. Subsequently, the ECA attention mechanism was integrated to realize the adaptive adjustment of the weights of different channels. Finally, depthwise separable convolution was utilized to fully integrate and classify the deep extraction of temporal and spatial features. In 10-fold cross-validation, the results show that AMANet achieves classification accuracies of 84.06 and 85.09% on the BCI Competition IV Datasets 2a and 2b, respectively, significantly outperforming baseline models such as Incep-EEGNet. On the High-Gamma dataset, AMANet attains a classification accuracy of 95.48%. These results demonstrate the excellent performance of AMANet in motor imagery decoding tasks.

## 1 Introduction

Brain–Computer Interface (BCI) is utilized to enable communication between the human brain and external devices without direct muscular or neural intervention (Pfurtscheller and Neuper, 2001; McFarland and Wolpaw, 2011). Among these, Motor Imagery BCI (MI-BCI) has been recognized forits noninvasiveness and high temporal resolution (Graimann et al., 2009). By acquiring neural signals in real time and decoding user intent from neuronal activation patterns (Pfurtscheller et al., 2006), BCI can control external devices (Ang et al., 2012, 2015) or facilitate information transfer (Li et al., 2010). Accurate decoding of electroencephalogram (EEG) signals generated during motor imagery not only underpins intelligent control of prostheses and robots but also holds promise for applications in medical rehabilitation and human–machine interaction (Condori et al., 2016; Cho et al., 2018). However, the complexity of EEG acquisition and the limited sample sizes constrain decoding performance (Craik et al., 2019), making enhancement of decoding accuracy under small-sample conditions critical for BCI applications.

In recent years, to address the challenge of small-sample EEG decoding, various convolutional neural network (CNN) (Lecun et al., 1998)-based models have been proposed by researchers to achieve efficient feature extraction and classification under limited-sample conditions. For example, Mattioli et al. combined a one-dimensional CNN (1D-CNN) (Mattioli et al., 2021) with the Synthetic Minority Oversampling Technique (SMOTE) (Chawla et al., 2002), increasing the overall classification accuracy in a five-class task from only 33.38% without augmentation to 99.38% after augmentation. Zhang et al. (2020) employed a generative model-based data augmentation approach in motor imagery tasks and performed classification using a single-scale CNN, achieving a substantial improvement in recognition accuracy with limited training data. Huang and Zhou (2021) proposed a hybrid approach that combines common spatial pattern (CSP) (Ramoser et al., 2000) with deep learning: CSP was first applied to enhance the spatial separability between two motor imagery classes, and the filtered signals were then fed into a deep network for end-to-end classification. It is noteworthy that this method focuses on enhancing feature separability rather than expanding the training dataset; thus, in sample-limited scenarios, further improvements in generalization performance can often be achieved by integrating such methods with data augmentation. Although effectiveness in small-sample environments has been shown by these approaches, they typically rely on single-scale convolution with a fixed receptive field, making it difficult to fully capture multi-scale time–frequency features (Zhang et al., 2021).

With the advancement of deep learning, multi-scale convolution structures (Ma et al., 2020) have been proposed by reasearchers to overcome the limitations of single-scale models in feature extraction and to enhance the capture of EEG information across different time–frequency scales. For example, Wu et al. developed MSFBCNN (Wu et al., 2019), which employs multiple parallel temporal convolution branches to extract multi-scale temporal features, combined with spatial convolution, thereby improving MI classification accuracy and cross-subject generalization on the BCI Competition IV dataset. Liu et al. designed FBMSNet (Liu et al., 2023), which integrates filter banks with multi-scale depthwise convolution to jointly extract frequency-band features, achieving approximately 79% accuracy across multiple datasets. Li et al. (2023) proposed MTFB-CNN, which applies multi-scale time–frequency block convolution directly to raw EEG signals without complex preprocessing, significantly improving MI decoding performance. While these methods enhance feature capture capability, they lack adaptive mechanisms to emphasize salient information and suppress noise, which limits decoding performance in small-sample scenarios.

To enhance EEG decoding performance, attention mechanisms have been gradually recognized as a key technique. They selectively emphasize critical information while suppressing irrelevant or redundant data, thereby improving feature representation (Ou and Zou, 2025). In recent years, attention mechanisms have been increasingly integrated into deep learning frameworks to highlight crucial information in motor imagery signals and boost classification performance. For example, CIACNet, was proposed by Liao et al. (2025) in which an improved CBA (Woo et al., 2018) module was incorporated and a dual-branch CNN with a temporal convolutional network (TCN) (Dudukcu et al., 2023) was combined to achieve multi-level feature extraction and fusion for MI-EEG signals, attaining excellent performance on the BCI-IV 2a and 2b datasets. Similarly, Altaheri et al. (2023) introduced ATCNet, integrating multi-head attention into the TCN structure to emphasize the most discriminative features, thus significantly enhancing decoding accuracy.

Inspired by these studies, AMANet, a data-augmented multi-scale temporal attention convolutional network for effective EEG decoding is proposed. The model is composed of five modules: a data augmentation block (DG-Block), a multi-scale temporal block (MST-Block), an ECA attention block, a depthwise separable convolution block (DSC-Block), and a classification block. The main contributions of this work are as follows:

- The AMANet model is proposed, which adaptively and dynamically captures small-sample EEG features and achieves superior results on the BCI Competition IV Dataset 2a and 2b.
- To address the limited sample size, a sliding window strategy is applied by this study to expand the training set and employs CSP to extract six discriminative spatial filter pairs, enhancing spatial feature extraction while reducing model parameters and improving robustness.
- To improve decoding performance, multi-scale temporal convolutions are employed for dynamic EEG feature extraction and pointwise convolution is adopted for channel integration, thereby strengthening multi-scale representation and enabling the model’s generalization to complex EEG signals to be improved.
- To emphasize critical channels and reduce computational cost, the efficient channel attention (ECA) mechanism is introduced, which highlights key features through adaptive weighting and enables lightweight spatial feature extraction without complex dimensionality reduction.

The remainder of this paper is organized as follows. The architecture of AMANet is introduced in Section 2. The experimental setup is described in Section 3. The experimental results are presented and discussed in Section 4, and finally, a brief conclusion is provided in Section 5.

## 2 Methods

**Figure 1:**
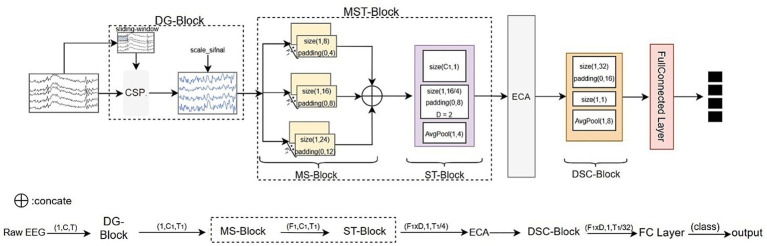
The overall structure of AMANet.

The architecture of AMANet is depicted in Figure 1. First, the training set is expanded and robustness is enhanced by the data augmentation block (DG Block). Next, both temporal and spatial features are extracted by the multi-scale temporal block (MST-Block)—comprising the multi-scale temporal feature block (MS-Block) and the spatial feature refinement block (ST-Block). Then, channel features are adaptively reweighted by An Efficient Channel Attention (ECA) mechanism to highlight the most discriminative information. Subsequently, spatiotemporal information is coupled by the DSC-Block for integrated feature representation. Finally, the fused features are assigned to the target classes by the classification layer assigns. Notably, to accommodate the multi-scale, non-stationary, and low signal-to-noise characteristics of EEG signals, this study adopts a multi-stage temporal convolutional architecture. The convolutions at different stages serve hierarchical temporal modeling functions rather than mere repetitive stacking. Specifically, the first-stage multi-scale convolution module extracts coarse-grained multi-temporal-scale features using kernels of different lengths, which can capture short-term local dynamics, medium-range rhythmic variations, and longer-term dependencies, acting as a learnable multi-band filter bank. The subsequent second-stage convolutions further integrate these coarse features in a fine-grained temporal manner and combine them with spatial filtering, achieving inter-channel feature projection and temporal enhancement. The third stage then strengthens the temporal features from the first two stages and suppresses noise through depthwise separable convolutions and lightweight channel attention, thereby improving the overall stability of feature representation.

To ensure consistent tensor dimensions, each convolutional layer employs padding tailored to its kernel size. The model parameters are listed in Table 1. With the exception of the CSP block, ELU activations are used, by which ReLU “dead neuron” issues are avoided and gradient flow is enhanced. Within the ECA mechanism, channel weights are normalized to the (0, 1) range by a sigmoid function preventing extreme weights from biasing feature representation.

**Table 1:** Hyperparameters of the AMANet model.

Block	Layer	Parameters	Output shape
Input			1, C , T
SW		window_size = 500, stride = 125	1, C , $T_{1}$
CSP		n_components= $C_{1}$	1, $C_{1}$ , $T_{1}$
scale		β∈(0.9,1.1)	1, $C_{1}$ , $T_{1}$
MS-Block	Conv2D (1)	size = (1,8), padding = (0, 4), stride = 1,	$F_{1}$ , $C_{1}$ , $T_{1}$
	BatchNorm2d	features= $F_{1}$ ,	$F_{1}$ , $C_{1}$ , $T_{1}$
	Conv2D (2)	size = (1,16) padding = (0,8), stride = 1	$F_{1}$ , $C_{1}$ , $T_{1}$
	BatchNorm2d	features= $F_{1}$ ,	$F_{1}$ , $C_{1}$ , $T_{1}$
	Conv2D (3)	size = (1,24) padding = (0,12), stride = 1	$F_{1}$ , $C_{1}$ , $T_{1}$
	BatchNorm2d	features= $F_{1}$	F1, $C_{1}$ , $T_{1}$
ST-Block	Conv2D(1)	size = ( $C_{1}$ ,1), groups= $F_{1}$ , stride = 1	$F_{1}*D$ ,1, $T_{1}$
	BatchNorm2d	features= $F_{1}*D$	$F_{1}*D$ ,1, $T_{1}$
	Conv2D(2)	size = (1, $K_{1}$ /4), padding = (0, $K_{1}$ /8), stride = 1	$F_{1}*D$ ,1, $T_{1}$
	BatchNorm2d	features= $F_{1}*D$	$F_{1}*D$ ,1, $T_{1}$
	AvgPool2d	size = (1,4), stride = 4	$F_{1}*D$ ,1, $T_{1}$ /4
ECA- Block		size = (3,3)	$F_{1}*D$ ,1, $T_{1}$ /4
DSF-Block	Conv2D(1)	size = (1, $K_{2}$ ), groups= $F_{1}*D$ padding = (0, $K_{2}$ /2), stride = 1	$F_{1}*D$ ,1, $T_{1}$ /4
	Conv2D(2)	size = (1,1), stride = 1	$F_{1}*D$ ,1, $T_{1}$ /4
	BatchNorm2d	features= $F_{2}$	$F_{1}*D$ ,1, $T_{1}$ /4
	AvgPool2d	size = (1,8), stride = 8	$\;F_{1}*D$ ,1, $T_{1}$ /32
Classifier	Linear1		32
	Linear2		4
Output			4

### 2.1 Data augmentation block

**Figure 2:**
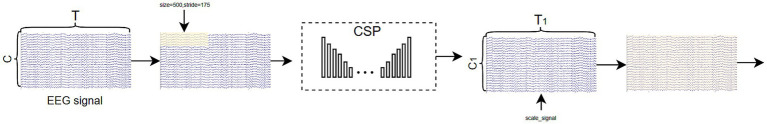
Schematic of the data augmentation block.


As illustrated in Figure 2, the block first applies a sliding window strategy with a window size of 500 and a stride of 75 to the EEG signals (with T sampling points), resulting in five overlapping sub-windows. The main reason for adopting this commonly used high-overlap strategy (Hwang et al., 2023) is that it can significantly increase the number of data samples while enhancing the model’s robustness and generalization ability without compromising temporal resolution, thereby providing richer training data for subsequent classification tasks. The original feature representation is transformed by The sliding window from $X\in {\mathbb{R}}^{C\times T}$ to $X_{1}\in {\mathbb{R}}^{C\times T_{1}}$ . Subsequently, the CSP algorithm is employed to extract spatial features from the segmented signals. To prevent data leakage between the training and test sets, CSP spatial filters are strictly computed on the training set and then applied only to the corresponding test set, ensuring that information from the test data is not utilized in the estimation of spatial filters. The normalized covariance matrices of different classes are computed by CSP, and generalized eigenvalue decomposition is performed to jointly diagonalize the matrices, thereby extracting the most discriminative spatial filters. The top six pairs of filters are selected by the algorithm to produce the output $X_{2}\in {\mathbb{R}}^{C_{1}\times T_{1}}$ . Finally, to enhance the model’s robustness to amplitude variations, the EEG signals are further subjected to linear scaling, which simulates inter-trial amplitude differences without altering the time–frequency structure. This procedure not only augments the training data and mitigates overfitting but also improves the model’s robustness to amplitude fluctuations, as shown in Equation 1:

$$X_{\text{augmented}}=\beta .X_{2}$$ (1)

Where β denotes the scaling factor. It follows a normal or uniform distribution (default value: 0.1), and is used to perturb the signal amplitude within a range of 90 to 110%. The augmented signals are then fed into the subsequent network to further extract spatiotemporal features, and classification performance is improved.

### 2.2 Multi-scale temporal block

**Figure 3:**
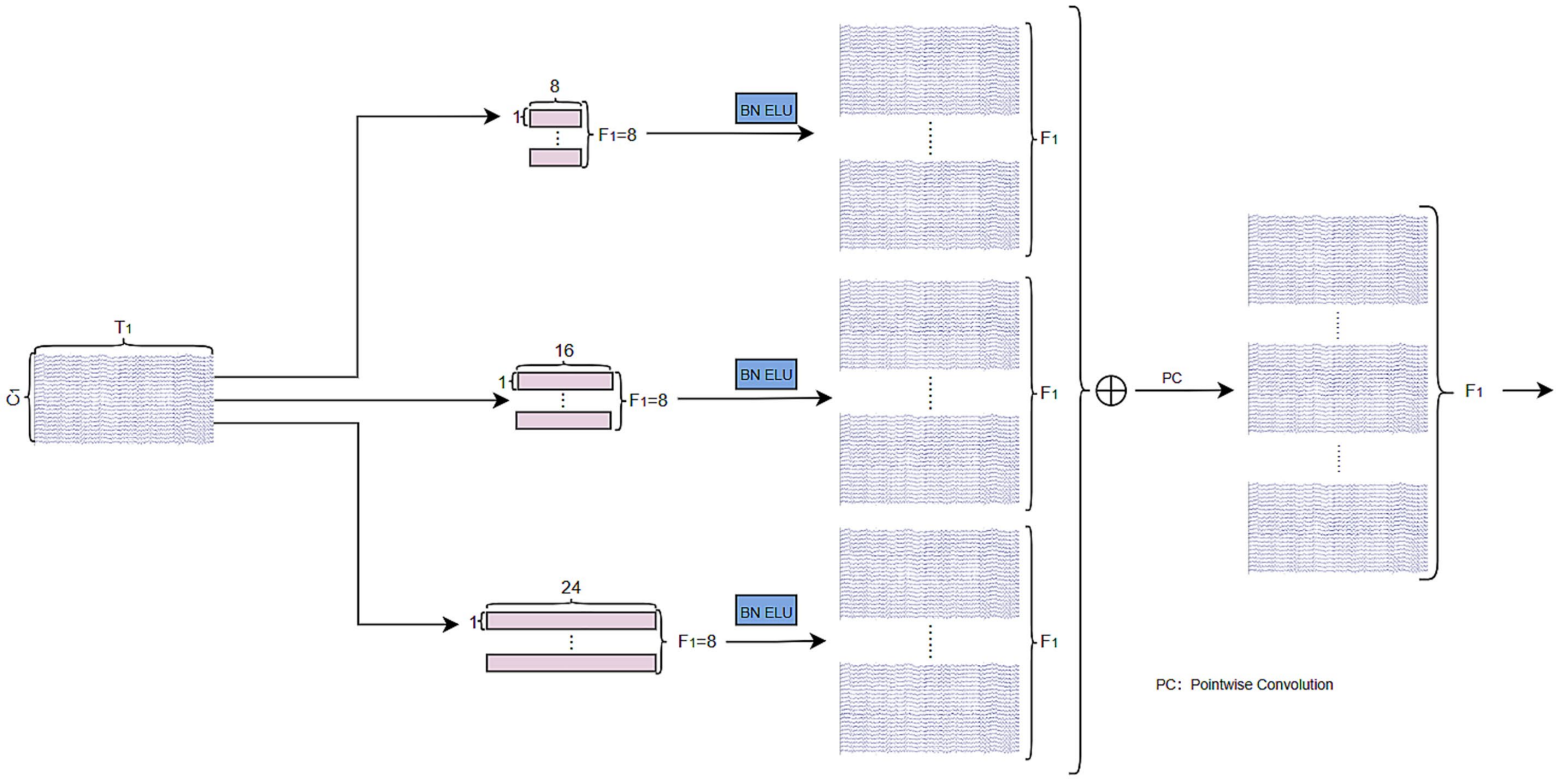
Multi-scale temporal convolutional structure.

The MST-Block is composed of the MS-Module and the ST-Module. Primarily designed to dynamically extract multi-scale temporal features from EEG signals (Tao et al., 2024; Chang et al., 2025) the MS- Block is as illustrated in Figure 3. According to previous studies (Lawhern et al., 2018; Ingolfsson et al., 2020), smaller convolutional kernels are more effective in capturing the temporal characteristics of EEG data. Therefore, the MS-Module employs three temporal convolutional layers with kernel sizes of (1, 8), (1, 16), and (1, 24), each of which comprises $F_{1}$ filters. The resulting feature tensor is denoted as ${X^{1}}_{\text{augmented}}\in {\mathbb{R}}^{F_{1}\times C_{1}\times T_{1}}$ . Each convolution is followed by batch normalization (BN) and the ELU activation function to enhance model stability and representational capacity. The outputs of the three layers are fused along the channel dimension, preserving the temporal integrity while reducing computational costs. The final output tensor remains ${X^{1}}_{\text{augmented}}\in {\mathbb{R}}^{F_{1}\times C_{1}\times T_{1}}$ . This module effectively captures the multi-scale temporal features of EEG data, which lays a solid foundation for subsequent spatial modeling and deep feature extraction.

**Figure 4:**
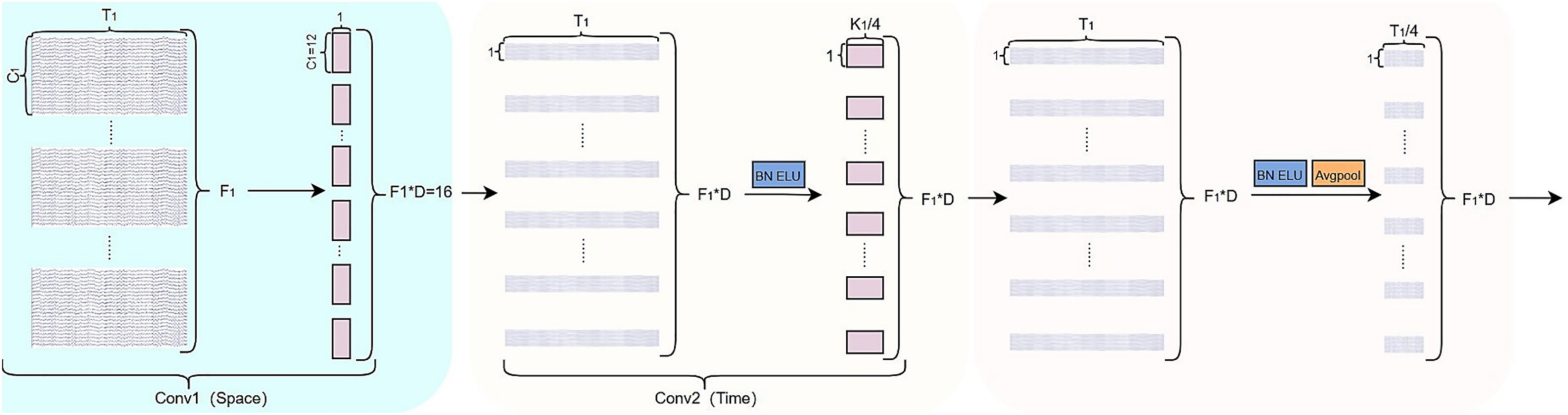
Spatial feature refinement block structure.

Designed to extract spatial features from EEG signals and then apply further temporal convolution, The ST- Block is as shown in Figure 4. It is composed of two consecutive convolutional layers, each followed by BN and an ELU activation. The first layer performs a global spatial convolution using D = 2 spatial filters of size $\left(C_{1},1\right)$ , where $C_{1}$ denotes the number of CSP feature channels. By setting $\text{groups}=F_{1}$ , each filter operates on a single channel to learn inter-channel relationships without mixing information. Its output feature map is represented as ${X^{2}}_{\text{augmented}}\in {\mathbb{R}}^{F_{1}*D\times C_{1}\times T_{1}}$ . The second layer applies a temporal convolution with kernel size $\left(1,K_{1}/4\right)$ to capture fine-grained time-domain features. Subsequently average pooling is performed along the temporal axis to reduce feature-map dimensions, decrease computational cost, suppress noise, and enhance robustness. The final output is ${X^{3}}_{\text{augmented}}\in {\mathbb{R}}^{F_{1}*D\times 1\times T_{1}/4}$ . Additionally, Dropout is introduced to mitigate overfitting.

### 2.3 Efficient channel attention block

**Figure 5:**
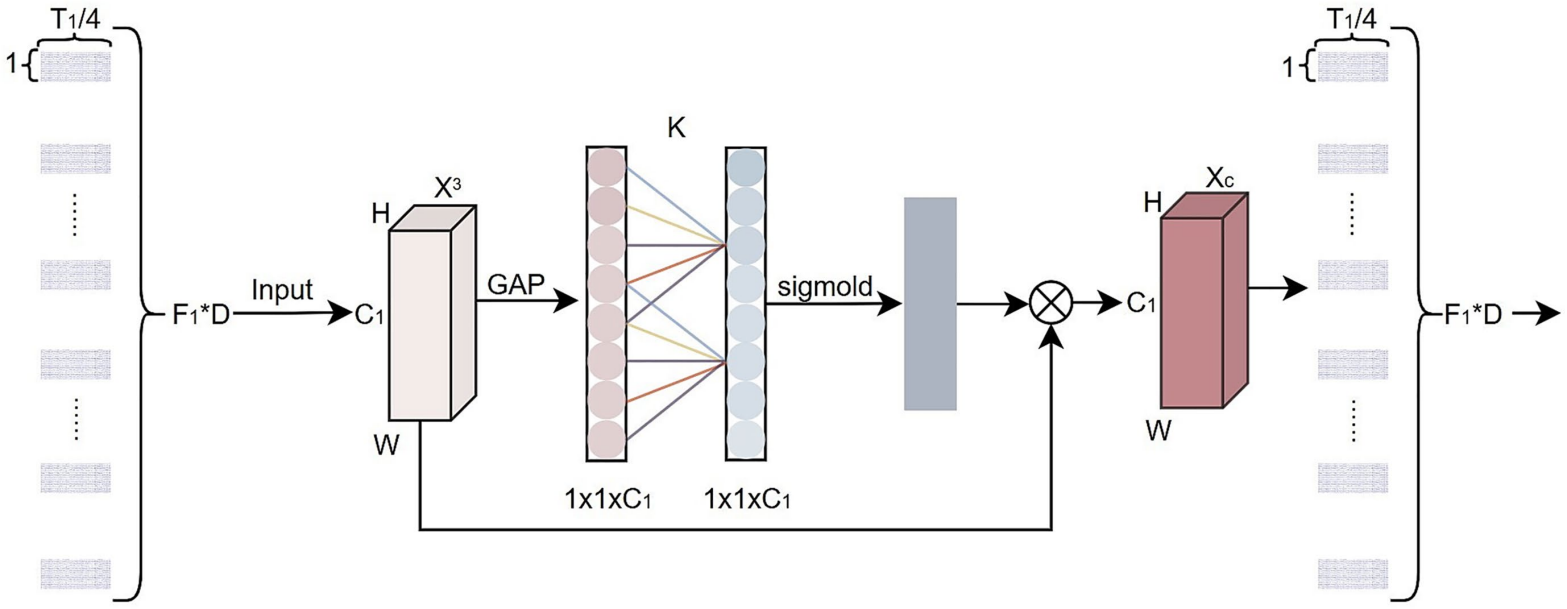
ECA block structure, Were, $C_{1}=12\text{or}2,H=1$ , $W=T_{1}/4=125$ , and K=3 denotes the convolutional kernel size. $X^{3}$ represents the input feature map, GAP denotes the global average pooling layer, and $X_{c}$ represents the output feature map.


ECA (Jia et al., 2023) is a lightweight yet effective channel attention mechanism that is designed to model inter-channel dependencies, enabling the network to focus on more discriminative feature channels. In this study, the design principle of ECA is functionally similar to that of self-attention mechanisms, as both enhance discriminability by adaptively weighting latent feature dimensions (Du et al., 2022). However, ECA achieves this in a more lightweight manner, making it suitable for small-sample EEG tasks. It should be noted that ECA is applied to the latent feature channels obtained after CSP spatial filtering and convolutional feature mapping. At this stage, each channel primarily reflects a combined representation of different spectral response patterns and spatial projection characteristics. The channel dimension no longer follows an explicit physical adjacency order, instead, it emphasizes the functional encoding of discriminative features. Therefore, in this work, ECA is not introduced based on an assumption of spatial adjacency. Rather, it is regarded as a lightweight adaptive weighting mechanism that leverages the latent relationships among features to emphasize important channels, suppress redundant channels, and highlight feature representations with higher discriminative contribution. As shown in Figure 5, different from the Squeeze-and-Excitation (SE) (Zhu et al., 2023) module, ECA replaces the fully connected layers with a 1 × 1 convolution, thus reducing parameter complexity without sacrificing performance. Specifically, the output feature map ${X^{3}}_{\text{augmented}}\in {\mathbb{R}}^{F_{1}*D\times 1\times T_{1}/4}\;$ of ST-Module is fed into the ECA module. First, global average pooling (GAP) compresses each features spatial dimensions (H × W) into a single scalar $z_{c}$ , as shown in Equation 2:

$$z_{c}=(\sum_{i}^{H}\sum_{j}^{W}{X^{3}}_{\text{augmented}})/(H\times W)c=1,2,\cdots C_{1}$$ (2)

Subsequently, a feature vector $z=[z_{1},z_{2}\cdots z_{C_{1}}]$ is obtained. A 1D convolution with a small kernel ( k=3 )is then applied along the channel dimension to capture local spatial features dependencies. The kernel size is calculated as shown in Equation 3:


$$\begin{array}{c} k=|(\log_{2}(C_{1})+b)/\gamma |\gamma =2b=1 \end{array}$$ (3)

The convolution result is subsequently normalized via a sigmoid activation function to generate channel-wise weighting coefficients, as defined in Equation 4:


$$\begin{array}{c} s=\sigma (\text{Conv}1D_{k}(z)),\sigma =\text{Sigmoid} \end{array}$$ (4)

Finally, the weighting coefficients are multiplied in a channel-wise manner with the original input features to achieve weighted feature optimization, yielding a final output $X_{c}\in {\mathbb{R}}^{F_{1}*D\times 1\times T_{1}/4}$ , as shown in Equation 5:


$$\begin{array}{c} X_{c}=s_{c}.{X^{3}}_{\text{augmented}},c=1,2,\cdots C_{1} \end{array}$$ (5)

The ECA module is introduced subsequent to the ST-Module to enhance the model’s focus on spatial features, thereby optimizing the feature representations fed into the DSF-Module and further improving overall classification performance.

### 2.4 Depthwise separable fusion block

**Figure 6:**
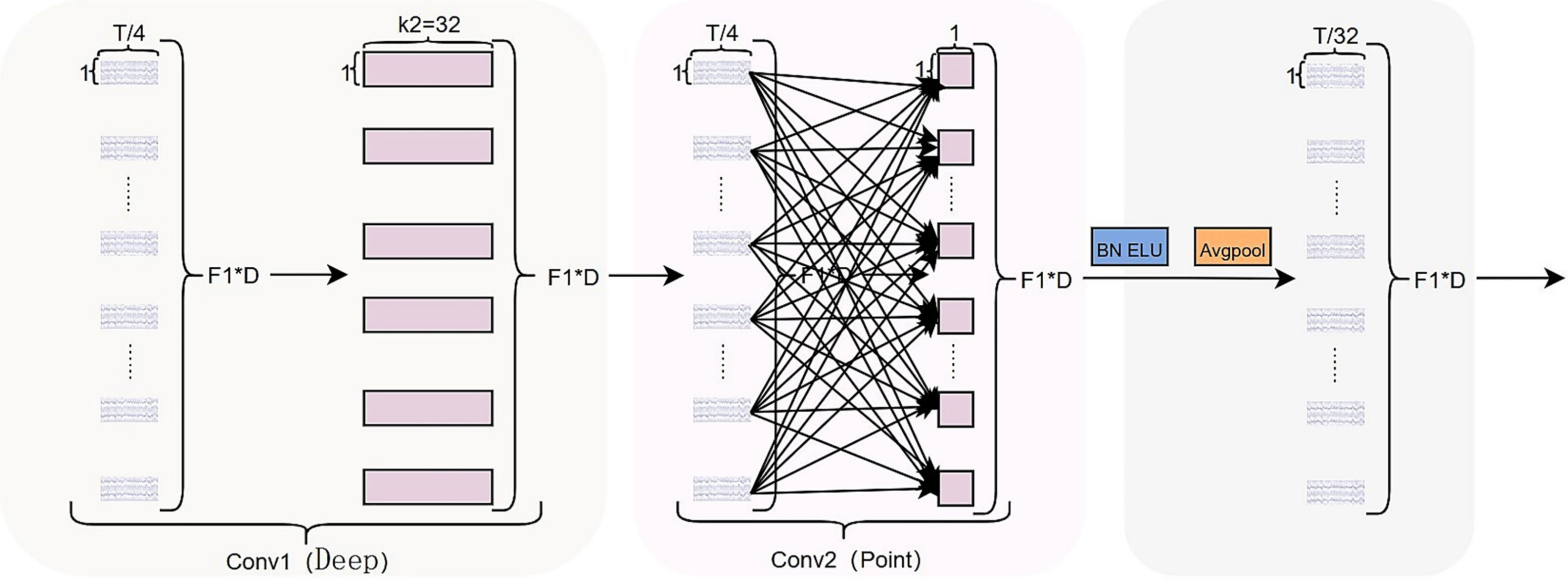
Depthwise separable fusion block structure.

The DSF-Block is devised to extract deeper temporal features. Its first layer is a depthwise convolution that performs temporal convolution independently on each input channel to prevent mixing of inter-channel information. Unlike the spatially focused depthwise convolution in the FSR-Module, the DSF-Module emphasizes deep temporal feature mining, Thus, setting groups = $F_{1}\times D$ ensures each filter operates only on its corresponding channel’s time series. The second layer is a pointwise convolution with a 1 × 1 kernel to fuse information across channels and enhance feature representation. An average pooling layer is also introduced to reduce parameter count. The final output feature map is represented as $X_{c}\in {\mathbb{R}}^{F_{1}*D\times 1\times T_{1}/32}$ . The architecture of the DSF-Module is shown in Figure 6. Through this meticulously designed hierarchy, the DSF-Module efficiently extracts deep temporal features and fully integrates inter-channel information, providing critical support for the model’s final classification performance.

### 2.5 Classification block

In the classification block, the input features are flattened to a vector whit a dimension $\text{of}\;F_{2}\times (T_{1}/32)$ . First they pass through a fully connected layer that reduces this to $F_{2}\times 2$ , followed by an ELU activation to introduce nonlinearity and enhance representational capacity. Dropout is then applied to mitigate overfitting and improve generalization. A second fully connected layer maps the $F_{2}\times 2$ features to the final number of classes, thus yielding the decision logits. Instead of explicitly applying Softmax, these logits are directly fed into PyTorch’s CrossEntropyLoss, which internally combines LogSoftmax and NLLLoss, to compute the classification loss in a numerically stable and streamlined manner.

## 3 Experiments

### 3.1 Dataset

To comprehensively evaluate the performance of the proposed network, this study utilize two motor imagery EEG datasets from BCI Competition IV, namely Dataset 2a and Dataset 2b.

Dataset 2a (Brunner et al., n.d.) includes EEG recordings from 9 subjects with 25 channels, which are band-pass filtered between 0.5–100 Hz. Each subject completed two sessions on different days, each containing 288 eight-second trials involving motor imagery of the left hand, right hand, both feet, or tongue. For analysis, EEG segments from 2 to 6 s after the cue were selected to capture stable task-related activity. Three EOG channels (left, right, center) were removed, thus leaving 22 EEG channels for classification.

Dataset 2b (Leeb et al., n.d.) also involves EEG recordings from 9 subjects, with only three channels: C3, Cz, and C4. The signals were sampled at 250 Hz and underwent band-pass filtering between 0.5 and 100 Hz. The experimental task involved motor imagery of the left and right hands, which comprised two non-feedback sessions and three feedback sessions. Each non-feedback session included 6 blocks per hand, with 10 trials per block, thus totaling 120 trials. Each feedback session consisted of 4 blocks per hand, with 20 trials per block, thus totaling 160 trials.

HGD dataset (Schirrmeister et al., 2017) consists of EEG recordings from 14 subjects acquired using 128 channels at a sampling rate of 500 Hz, with the signals band-pass filtered between 0.5 and 100 Hz and additionally processed with a 50 Hz notch filter to suppress power-line noise. The experimental paradigm involves four motor imagery tasks, corresponding to left hand, right hand, both feet, and tongue movements. Each subject completed four sessions, with 160 trials per session. In this study, EEG segments from 0.5 to 4.5 s after cue onset, corresponding to the stable task period, were selected for analysis, and all EEG channels were utilized for classification.

### 3.2 Training procedure

To evaluate the effectiveness of the AMANet model, this study adopted 10-fold cross-validation and utilized the Adam optimizer for model training. The initial learning rate was set to 0.001, the batch size was set to 32, and the dropout rate was set to 0.3. Taking the BCI Competition IV-2a dataset as an example, the data of each subject was partitioned into 10 subsets. In each round, one subset served as the validation set, whereas the remaining nine subsets were used for training. This procedure was iterated ten times. The adoption of 10-fold cross-validation not only maximizes data utilization but also helps prevent overfitting and enhances the model’s generalization capability. The random seed was fixed at 1234, and training was conducted for 300 epochs. Early stopping was not applied. The model was implemented in PyTorch and trained on an NVIDIA GeForce GTX 1660 Ti GPU.

## 4 Results and discussion

This study trains and evaluates AMANet on the four-class BCI Competition IV Dataset 2a, the binary Dataset 2b, and High-Gamma datasets, comparing its classification accuracy with that of baseline models. Results are visualized via confusion matrices. Ablation experiments are subsequently conducted to demonstrate the contributions of CSP and AMANet. Finally, key parameters, such as the number of temporal and spatial filters, are tuned to select the optimal classification model.

### 4.1 Classification accuracy

**Figure 7:**
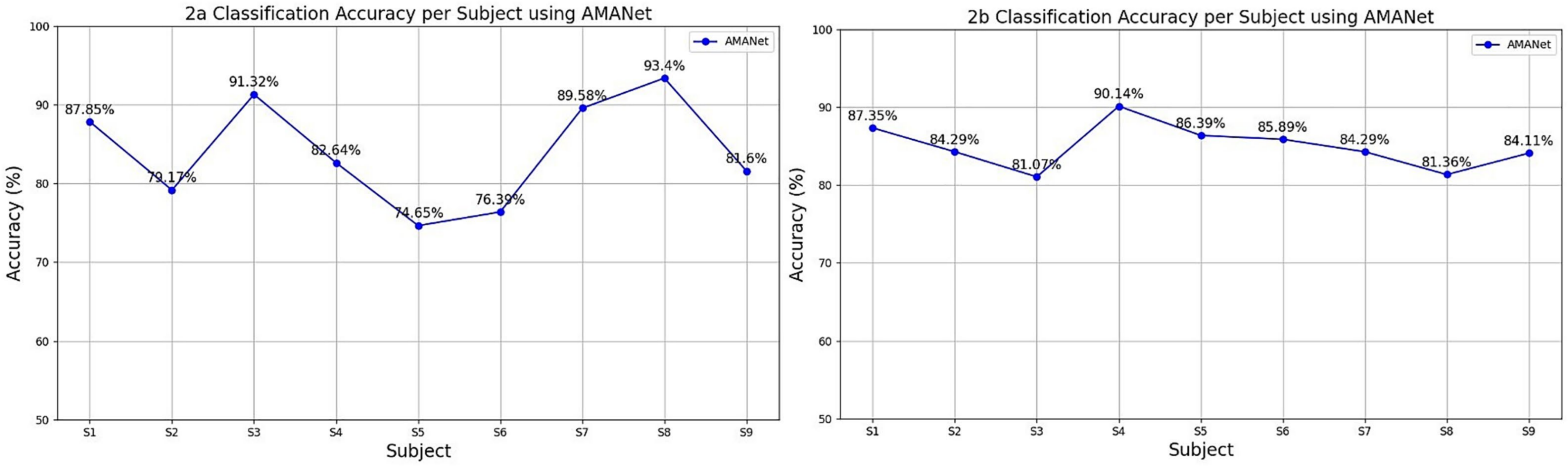
Classification results of different subjects in the IV-2a and IV-2b datasets.

As shown in Figure 7, the classification accuracies of the AMANet model for nine subjects (S1–S9) in the BCI Competition IV-2a and IV-2b datasets are presented. Overall, the model achieved an average accuracy of 84.06% on the IV-2a dataset. Notably, S3 (91.32%), S7 (89.58%), and S8 (93.40%) attained the highest accuracies, indicating a strong discriminative capability for these subjects, which may be attributed to more distinct signal features, higher data quality, or greater consistency in task execution strategies. In contrast, S5 (74.65%) and S6 (76.39%) exhibited comparatively lower performance, possibly owing to insufficient EEG samples or poorer signal quality. For the 2b dataset, accuracies also varied substantially across subjects, with S4 achieving the highest accuracy (90.14%) and S3 the lowest (81.07%). Overall, all subjects exceeded an accuracy of 80%, demonstrating the model’s strong recognition ability for most individuals. Nevertheless, these results also suggest that AMANet could be further optimized to better address inter-subject variability in the IV-2b dataset.

### 4.2 Model comparison

The classification accuracies and kappa values of our proposed AMANet and six benchmark models—EEGNet (Lawhern et al., 2018), Incep-EEGNet (Riyad et al., 2020), FBCSP (Ang et al., 2008), ShallowConvNet, DeepConvNet (Schirrmeister et al., 2017) and TCNet Fusion (Musallam et al., 2021)—on the BCI Competition IV 2a dataset are presented in Table 2. At the single-subject level, AMANet achieved the highest accuracy and *κ* on S2, S4, S6 and S8, with S8 reaching an accuracy of 93.40% and a κ of 0.91. Compared with the benchmarks, this corresponds to improvements of 4.48–13.85% in accuracy and 0.06–0.20 in κ. The exceptional result on S7 indicates strong subject-specific adaptability. In contrast, S5 and S6 recorded lower accuracies (74.65 and 76.39%) and κ values (0.66 and 0.68). This may be attribute to EEG noise or artifacts affecting feature extraction or to suboptimal parameter settings leading to overfitting. Notably, despite the relatively low performance of S6, AMANet still outperformed all benchmark models on that subject, further demonstrating its superiority. In addition except for the TCNet Fusion model, whose significance difference is greater than 0.05 (*p*-value), all other baseline models exhibit significance differences less than 0.05 (p-value). Overall, AMANet achieved a mean accuracy of 84.06% and a mean κ of 0.78—both exceeding those of the benchmark models—indicating superior classification consistency and stability.

**Table 2:** Comparison of classification accuracy (%) and kappa value (k) on the BCI Competition IV-2a dataset (4 classes).

Subject	EEGNet		Incep-EEGnet		FBCSP		ShallowConvNet		DeepConvNet		TCNet Fusion		proposed	
	Accuracy	k	Accuracy	K	Accuracy	K	Accuracy	K	Accuracy	K	Accuracy	K	Accuracy	K
S1	83.91	0.78	79.6	0.72	75.62	0.65	83.47	0.76	81.28	0.75	90.74	0.87	87.85	0.83
S2	66.49	0.55	57.72	0.44	57.6	0.47	58.21	0.46	59.52	0.45	70.67	0.60	79.17	0.72
S3	87.29	0.83	89.27	0.83	82.21	0.74	86.69	0.82	88.12	0.85	95.23	0.93	91.32	0.88
S4	59.39	0.46	65.69	0.57	62.09	0.48	64.17	0.43	63.88	0.51	76.75	0.68	82.64	0.76
S5	64.88	0.54	63.37	0.51	55.08	0.46	68.52	0.63	70.24	0.60	82.24	0.76	74.65	0.66
S6	60.59	0.48	56.12	0.47	41.76	0.31	55.73	0.56	64.82	0.51	68.83	0.58	76.39	0.68
S7	72.81	0.65	88.72	0.81	81.79	0.76	88.69	0.87	87.12	0.83	94.22	0.92	89.58	0.86
S8	81.06	0.74	82.76	0.78	79.67	0.71	79.45	0.76	82.14	0.77	88.92	0.85	93.40	0.91
S9	85.57	0.81	82.23	0.78	69.76	0.69	78.54	0.77	83.66	0.78	85.98	0.81	81.60	0.75
Average	73.55	0.64	73.94	0.65	67.2	0.58	73.71	0.67	75.64	0.67	83.73	0.78	**84.06**	**0.78**
P-value	0.004**		0.006**		0.0003**		0.005**		0.009**		0.71			

*p* < 0.05 indicates a statistically significant difference, while *p* ≥ 0.05P indicates no significant difference. Bold face indicates that the proposed method in this paper achieves the best performance.

Table 3 presents the classification results of the AMANet model on the BCI Competition IV-2b dataset, compared with five benchmark models: FBCSP (Ang et al., 2008), EEGNet (Lawhern et al., 2018), EEG-ITNet (Salami et al., 2022), 1D-Multi-scale-CNN (Tang et al., 2020) and SHNN (Liu et al., 2022). The results show that AMANet achieved the highest or near-optimal performance for most subjects. For instance, it attained peak accuracies of 87.35 and 84.29% for S1 and S2, respectively, significantly outperforming the compared models. For S9, it achieved 84.11%, slightly lower than that of 1D-Multi-scale-CNN (86.81%) but far higher than that of EEG-ITNet (55.31%). Although the accuracy of AMANet was marginally lower than EEG-ITNet and SHNN for certain subjects (e.g., S4 and S8), its overall performance remained stable and reliable. Notably, for subjects S3, S5, S6, and S7, where EEG-ITNet performed better, AMANet still maintained accuracies above 80%, which demonstrates strong robustness. Overall, AMANet achieved an average classification accuracy of 85.09% across all subjects, surpassing EEG-ITNet (82.59%) and SHNN (83.49%), thus validating its effectiveness and comprehensive advantage in motor imagery BCI classification tasks.

**Table 3:** Comparison of classification accuracy (%) on the BCI competition IV-2b dataset.

Subject	FBCSP	EEGNet	EEG-ITNet	1D Multi-scale CNN	SHNN	proposed
S1	70.00	67.50	67.50	80.56	83.33	87.35
S2	60.36	60.35	71.43	65.44	61.76	84.29
S3	60.94	62.81	86.88	65.97	58.33	81.07
S4	97.50	91.25	98.44	99.32	97.30	90.14
S5	93.12	83.44	94.06	89.19	91.89	86.39
S6	80.63	61.56	86.25	86.11	88.89	85.89
S7	78.13	83.75	90.00	81.25	86.11	85.29
S8	92.50	91.88	93.44	88.82	92.11	81.36
S9	86.88	82.50	55.31	86.81	91.67	84.11
Average	80.00	76.12	82.59	82.61	83.49	**85.09**

Bold face indicates that the proposed method in this paper achieves the best performance.

The classification accuracy and Kappa values of different models on the High-Gamma dataset are presented in Table 4. As show in the table the traditional FBCSP method yields the poorest performance, with an average accuracy of only 71.04% and an average Kappa of 0.62, whereas deep learning models significantly outperform traditional approaches. Specifically, ShallowConvNet, DeepConvNet, and TCNet Fusion achieve average accuracies exceeding 94% with Kappa values around 0.94, where demonstrates the strong capability of deep convolutional networks in extracting high-frequency motor imagery EEG features. EEGNet and Incep-EEGNet, as lightweight convolutional networks, also exhibit stable performance, with average accuracies of 89.67 and 93.03%, and corresponding Kappa values of 0.85 and 0.91. Notably, the proposed model in this study consistently achieves excellent performance across all subjects, with an average accuracy of 95.48% and a Kappa of 0.94. It attains the highest or near-highest classification performance for subjects such as S1, S2, and S5, highlighting its superior capability in capturing key high-frequency EEG features and its robust cross-subject stability. Overall, although various deep convolutional networks can effectively improve motor imagery EEG classification performance, the proposed model consistently achieves the best or near-best performance across subjects. This indicates that our model has stronger feature extraction ability and higher classification stability, thus significantly outperforming existing methods.

**Table 4:** Comparison of classification accuracy (%) and kappa values (k) on the high-gamma dataset.

Subject	EEGNet		Incep-EEGnet		FBCSP		ShallowConvNet		DeepConvNet		TCNet Fusion		proposed	
	Accuracy	k	Accuracy	K	Accuracy	K	Accuracy	K	Accuracy	K	Accuracy	K	Accuracy	K
S1	86.76	0.80	92.81	0.92	61.93	0.51	93.21	0.92	97.35	**0.97**	95.75	0.93	**98.31**	0.96
S2	90.34	0.81	94.69	0.91	68.82	0.58	95.47	0.94	96.87	0.95	98.32	0.95	**98.12**	**0.98**
S3	95.63	0.93	95.12	0.93	89.73	0.88	96.87	0.96	97.50	0.95	**98.98**	**0.98**	97.14	0.96
S4	93.6	0.92	94.38	0.92	86.23	0.86	98.35	0.96	**98.10**	**0.98**	94.17	0.91	96.54	0.95
S5	92.35	0.90	93.45	0.93	74.32	0.65	98.91	0.97	97.69	0.97	97.52	0.96	**98.32**	**0.97**
S6	91.23	0.87	94.05	0.88	66.05	0.58	96.21	**0.96**	**97.32**	0.95	95.63	0.95	95.35	0.92
S7	89.15	0.83	92.97	0.90	73.56	0.61	94.67	0.93	**95.62**	**0.95**	93.17	0.91	91.12	0.89
S8	90.47	0.88	94.53	0.96	73.58	0.69	98.12	0.97	93.17	0.91	95.67	0.94	**96.54**	**0.95**
S9	92.76	0.91	93.21	0.89	60.35	0.51	97.1	0.97	96.35	**0.96**	**98.06**	0.98	92.37	0.92
S10	88.45	0.85	**94.29**	**0.93**	63.21	0.53	91.42	0.88	92.47	0.91	90.21	0.88	91.39	0.89
S11	82.75	0.81	92.86	0.91	61.78	0.52	**98.82**	**0.98**	97.86	0.97	8,656	0.78	96.75	0.93
S12	93.82	0,92	92.64	0.92	82.19	0.76	94.79	0.95	96.89	0.94	94.35	0.93	**95.34**	**0.95**
S13	85.73	0.80	93.58	0.91	75.42	0.66	96.23	0.93	**98.72**	**0.98**	94.73	0.94	96.72	0.95
S14	82.34	0.79	83.82	0.81	57.35	0.47	81.2	0.75	77.35	0.71	88.29	0.83	**92.71**	**0.91**
Average	89.67	0.85	93.03	0.91	71.04	0.62	95.15	0.94	95.23	0.94	94.36	0.94	**95.48**	**0.94**

Bold face represents the best results of different models for different subjects in the dataset.

Table 5 presents a comparison between AMANet and other mainstream models on the IV-2a dataset in terms of trainable parameters, computational complexity (MACs), memory requirements, and classification accuracy. MACs (Multiply–Accumulate Operations) measure the total number of multiplication–addition operations during a single forward pass, while memory requirements refer to the total memory occupied by the output feature maps of all network layers (Li et al., 2023). Specifically, AMANet has 8.09 times more trainable parameters than EEGNet, yet still fewer than those of other compared models; its average MACs are 1.22 times higher than those of EEGNet, but remain lower than those of the other baselines. Notably, due to the increased network depth, its memory usage is increased by 17.34 times compared to EEGNet. These results indicate that AMANet achieves higher classification accuracy by enhancing the feature extraction capability at the cost of additional parameters, computational complexity, and memory consumption (Rizvi et al., 2023).

**Table 5:** Comparison of model complexity and performance on the BCI competition IV-2a dataset.

Model	Accuracy	Parameters	Average MACs	Memory requirement
EEGNet	72.99	2.63 K	13.1 M	396 KB
ShallowConvNet	73.71	47.3 K	63.0 M	1,013 KB
Riemannian (Hersche et al., 2018)	74.77	50.0 k	-	49 KB
FBCSP (Ang et al., 2008)	67.2	261 K	104 M	50 KB
MSFBCNN (Wu et al., 2019)	75.12	155 k	202 M	5,775 KB
proposed	**84.06**	21.3 K	16.1 M	6.87 MB

Bold face indicates that the proposed method in this paper achieves the best performance.

### 4.3 Visualization

**Figure 8:**
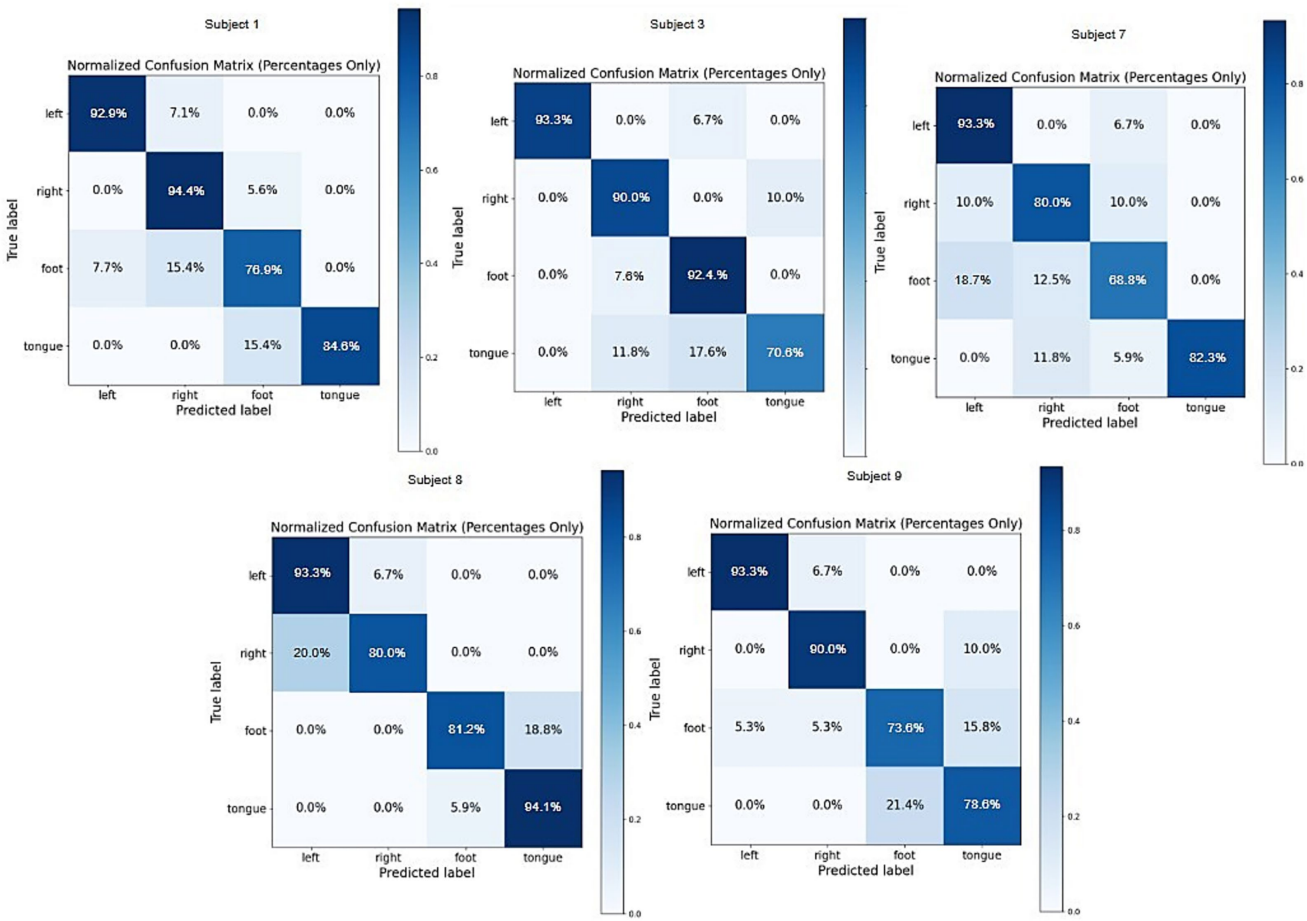
Confusion matrix of dataset 2a (S1, S3, S7, S8, S9).

This section employs confusion matrices and t-distributed stochastic neighbor embedding (t-SNE) to evaluate the model’s performance. The confusion matrices of the AMANet model for five subjects (S1, S3, S7, S8, S9) from the BCI Competition IV-2a dataset are presented in Figure 8. Each matrix includes the labels “left,” “right,” “foot,” and “tongue,” where diagonal entries represent correct classification rates and off-diagonal entries indicate misclassifications. The results demonstrate that subject S8 achieved the best overall performance, with the darkest diagonal and the lightest off-diagonal colors. Specifically, the accuracies for “tongue” and “left” were the highest, reaching 94.1 and 93.3%, respectively. The “foot” class achieved an accuracy of 81.2%, with 18.8% misclassified as “tongue,” this may stem from the overlapping spatial activation patterns of the two types of EEG signals. Previous studies have indicated that foot movements primarily activate the central sensorimotor area, whereas tongue movements are more associated with the lateral cortex (Pfurtscheller et al., 2006). However, individual differences may make it difficult for CSP features to achieve effective separation. The “right” class had the lowest accuracy of 80.0%, with 20.0% misclassified as “tongue,” which reflects the similarity of EEG features between left- and right-hand motor imagery. Overall, AMANet demonstrated superior performance on S8 in comparison with other subjects, suggesting its effectiveness in capturing individual EEG characteristics. Nevertheless, inter-subject variability remains, which may be attributed to the higher signal quality or more stable motor imagery features in S8. These findings highlight the potential of incorporating personalized training strategies for further enhancing the model’s generalization and robustness across individuals.

**Figure 9:**
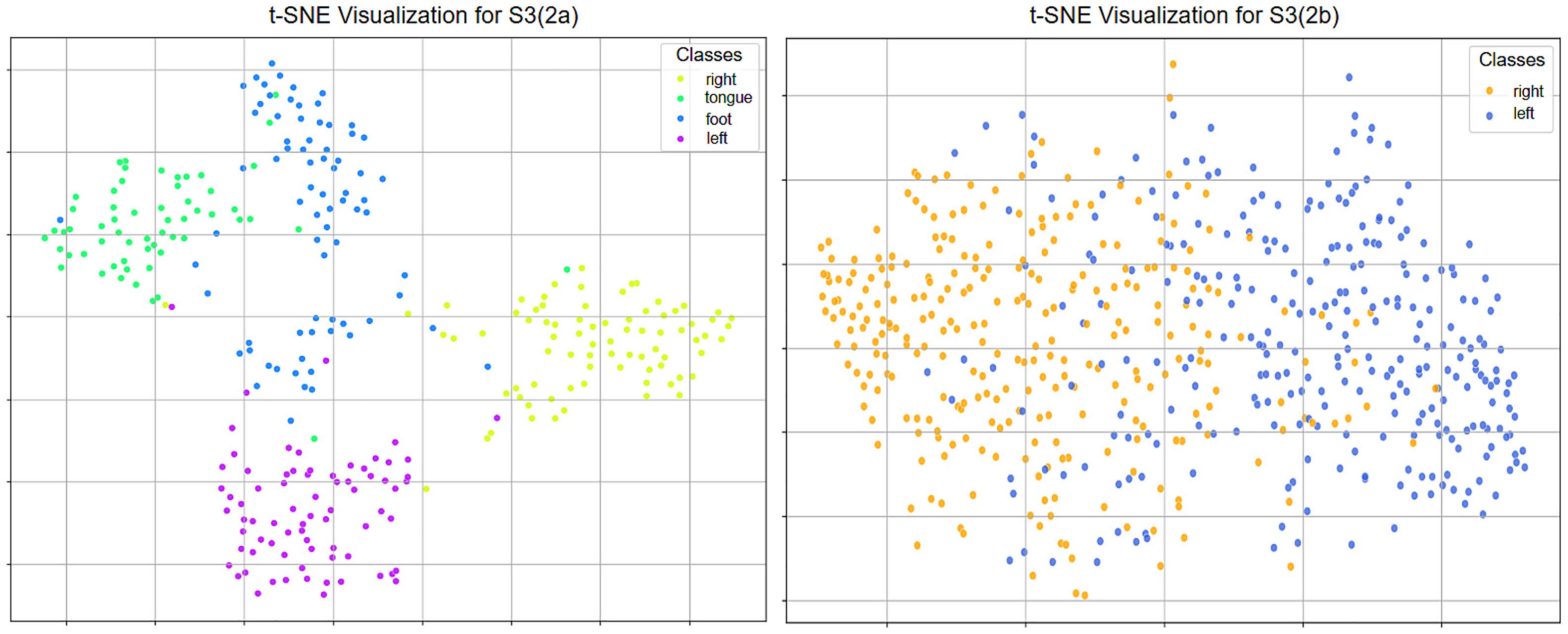
t-SNE distribution map of dataset S3 for 2a and 2b.

To further evaluate the proposed model’s performance in feature separability, this study employed the t-SNE method to visualize the distribution of different motor imagery samples in a low-dimensional space. The feature distribution of subject S3 from the BCI Competition IV 2a and 2b datasets is illustrated in Figure 9. The results indicate that for S3, the four-class motor imagery tasks (left hand, right hand, foot, and tongue) exhibit well-formed clusters with clear inter-class boundaries in the two-dimensional space, which demonstrates the model’s strong capability in feature extraction and multi-class discrimination. In contrast, the two-class tasks (left hand and right hand) for the same subject exhibit a certain degree of overlap, although relatively distinct boundaries can still be observed. Overall, the model demonstrates more prominent discriminative performance in multi-class tasks, while some room for improvement remains in binary tasks for certain subjects.

### 4.4 Ablation study

Ablation results forAMANet’s multi-scale module, attention mechanism, and data augmentation on Dataset 2a are reported in Table 6. With only attention mechanism and augmentation enabled—excluding the multi-scale module—the accuracy was 81.56%. Adding the multi-scale module further raises the accuracy to 84.06%, the highest value, which demonstrates its effectiveness in capturing discriminative features across temporal scales (Shen et al., 2022). Removing the attention mechanism (while retaining only the multi-scale module and augmentation) reduces the accuracy to 81.13%, indicating the critical role of the attention module in allocating channel weights to enhance key features. Omitting data augmentation (while using only the multi-scale module and attention) result an accuracy drop to 75.67%, underscoring the importance of data augmentation in mitigating overfitting and improving robustness in small-sample EEG scenarios. Further analysis of the roles of the ST-Block and DSF-Block shows that when the ST-Block is removed, leaving only the multi-scale module, attention mechanism, and DSF-Block, the accuracy drops to 77.39%, indicating that the ST-Block plays an important role in spatiotemporal feature extraction and in capturing long-term temporal dependencies, contributing significantly to model performance. Conversely, when the DSF-Block is removed, leaving only the multi-scale module, attention mechanism, and ST-Block, the accuracy decreases to 79.06%, demonstrating that the DSF-Block is also indispensable for spatial feature fusion and modeling feature dependencies. Overall, these results validate the synergistic contributions of the multi-scale module, attention mechanism, data augmentation, ST-Block, and DSF-Block in enhancing model performance, with each component making a substantial contribution to the final classification accuracy.

**Table 6:** Impact of different modules on the classification accuracy of the AMANet model.

Data enhancement	Multi-scale	Attention mechanism	ST-Block	DSF-Block	Accuracy (%)
√	×	√	√	√	81.56
√	√	√	√	√	84.06
√	√	×	√	√	81.13
×	√	√	√	√	75.67
√	√	√	×	√	77.39
√	√	√	√	×	79.06

**Figure 10:**
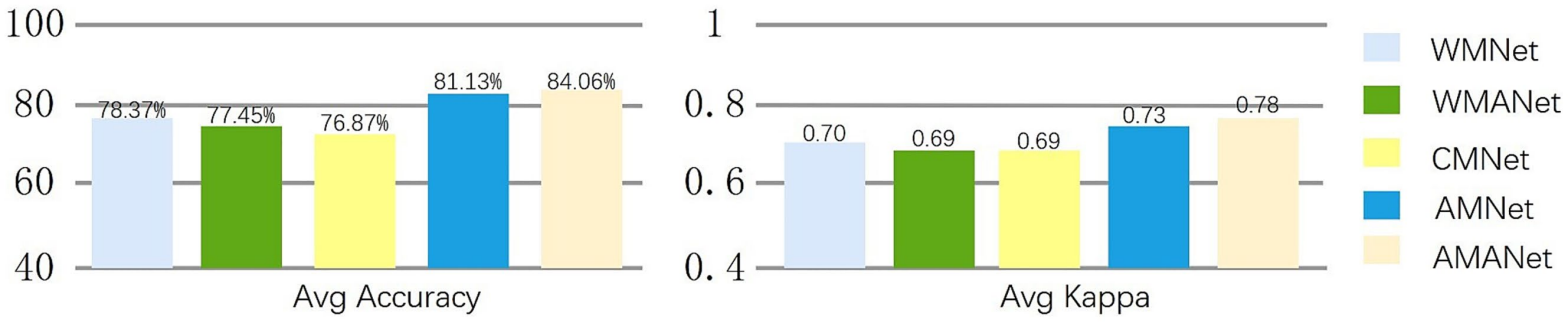
Classification accuracy and kappa values for different network structures.

To assess the specific effects of the CSP and ECA modules, five model variants (Figure 10) were designed. The baseline model, WMNet, which excludes both CSP and ECA, achieved an accuracy of 78.37% with a *κ* value of 0.70. When only the ECA module was added (WMANet), the performance decreased to 77.45% accuracy with a κ value of 0.69, indicating that without CSP providing discriminative spatial features, ECA may amplify irrelevant or redundant feature information, which is detrimental to classification. By contrast, CMNet, which includes CSP but does not use data augmentation, achieved 76.87% accuracy with a κ value of 0.69, lower than that of WMNet. This suggests that applying CSP alone, when training data diversity is insufficient, may lead to underfitting of specific spatial patterns in the training set, limiting generalization. When AMNet incorporated CSP while retaining data augmentation, performance improved substantially, achieving 81.13% accuracy with a κ value of 0.73. This demonstrates that data augmentation enhances the robustness of the discriminative spatial features extracted by CSP. Finally, the full model, combining CSP and ECA modules under data augmentation, achieved the best performance with 84.06% accuracy with a κ value of 0.78, indicating that CSP provides reliable spatial discriminative features while ECA performs fine-grained weighting on these features, resulting in a synergistic effect that significantly enhances overall classification capability.

### 4.5 Parameter optimization

#### 4.5.1 Selection the size of the convolutional kernel

**Figure 11:**
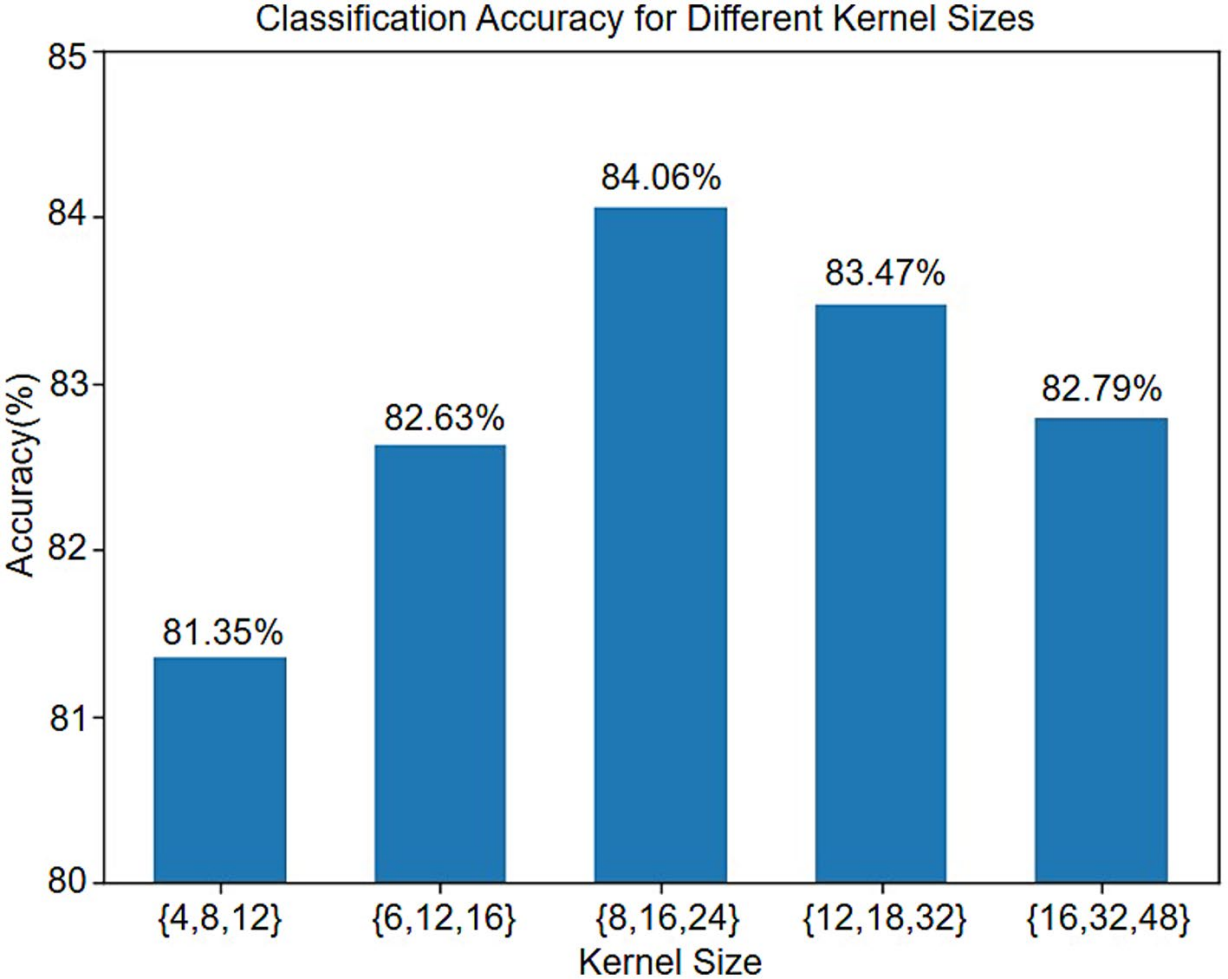
Classification results of different combinations of convolution kernels.

The impact of different convolutional kernel size combinations in the multi-scale temporal encoding module on motor imagery EEG classification accuracy is illustrated in Figure 11. On Dataset 2a, kernel selection proves critical: enlarging the kernel set from {4, 8, 12} to {8, 16, 24} increases the accuracy from 81.35 to 84.06%, demonstrating that a moderate kernel expansion broadens the receptive field and enhances the capture of temporal features. However, further enlarging to {12, 18, 32} and {16, 32, 48} reduces the accuracy to 83.47 and 82.79%, respectively—likely because overly large kernels, despite extracting wider temporal context, markedly increase the parameter count, complicate the training process, over-smooth features, obscure local details, and promote overfitting. Therefore, model design must balance the feature extraction capacity against complexity, with {8, 16, 24} identified as the optimal kernel combination.

#### 4.5.2 Attention mechanism selection

To analyze the impact of different attention mechanisms on classification performance, this study compared four settings: ECA, CBAM, SE, and No-Attention, with the experimental results summarized in Table 7. Overall, all three attention mechanisms achieved more stable performance improvements compared to the model without attention, confirming the effectiveness of incorporating attention mechanisms in EEG motor imagery classification tasks. For individual subject results, ECA achieved the best performance on S1, S3, and S8, demonstrating its strong feature enhancement capability for specific subjects. CBAM achieved the highest classification accuracy on S2, S5, and S7, with S7 reaching 93.10%, the highest among all subjects, indicating that the simultaneous application of channel and spatial attention helps to effectively model the local saliency distribution of EEG features. SE slightly outperformed other methods on S3 and S9, reflecting its advantage in modeling channel-wise dependencies under certain scenarios. In contrast, the No-Attention model generally exhibited relatively lower performance across most subjects. Regarding overall average performance, ECA achieved the highest mean classification accuracy of 84.06%, with a Kappa coefficient of 0.78, which is also superior to CBAM and SE (both 0.76) and the No-Attention model (0.73). This indicates that ECA offers superior stability and overall generalization. The underlying reason may be that SE recalibrates channel weights through global average pooling and fully connected layers, and while it can model channel dependencies, it may lose some fine-grained discriminative information during the “squeeze-and-excitation” process. CBAM, by incorporating both channel and spatial attention, can highlight local salient features more precisely but features a more complex structure, which increases the risk of overfitting in small-sample EEG scenarios. In contrast, ECA enables local interactions along the channel dimension using lightweight one-dimensional convolutions, effectively preserving fine-grained feature information without dimensional compression. This not only reduces model complexity but also improves overall stability and generalization. Considering accuracy, Kappa coefficient, and model complexity, ECA was ultimately selected as the attention module for AMANet, ensuring high performance while maintaining computational efficiency and model robustness.

**Table 7:** Comparison of different attention mechanisms.

Subject	ECA	CBMA	SE	No Attention
S1	87.85	82.78	86.57	84.87
S2	79.17	80.94	76.72	74.15
S3	91.32	89.91	91.66	89.62
S4	82.64	75.32	77.28	75.67
S5	74.65	78.56	72.28	71.21
S6	76.39	72.57	75.77	74.15
S7	89.58	93.1	91.32	88.92
S8	93.40	88.64	88.57	86.74
S9	81.60	83.47	84.68	84.85
Average	84.06	82.81	82.76	81.13
K	0.78	0.76	0.76	0.73

#### 4.5.3 Sliding window selection

To analyze the impact of the sliding window approach on experimental results, the classification accuracy under several conditions was compared in this study: without a sliding window, and with a window length of 500 and step sizes of 250, 125, 75, and 25, respectively. The results are presented in Table 8. Without the application of a sliding window, the average accuracies on the 2a and 2b datasets were 75.85 and 76.14%, respectively. After the employment of the sliding window method, the lowest average accuracies increased to 78.15 and 78.45%, which indicates that the method not only enlarged the sample size but also significantly improved the classification performance. Notably, when the step size was set to 75, the average accuracies of the nine subjects in the two datasets reached 84.06 and 85.09%, achieving the best performance. However, when the step size was further reduced to 25, the accuracies dropped by 2.83 and 1.24%, respectively. This decline may be attributed to excessive overlap in the data, which made the model more sensitive to redundant information and thus negatively affected classification outcomes.

**Table 8:** Classification accuracy (%) at different sliding window time intervals.

dataSet	Not use	(500, 250)	(500, 125)	(500, 75)	(500, 25)
2a	75.85	78.15	80.82	**84.06**	81.23
2b	76.14	78.45	81.25	**85.09**	83.85

Bold face indicates the best performance.

Next, the sliding interval to 75, with window lengths of 750, 625, 500, 375, and 250 was set, respectively. As shown in Table 9, when the sliding window length is 500, the average classification accuracy of the two datasets reaches the highest values of 84.06 and 85.09%, which achieves optimal classification performance. Therefore, the sliding window is set to 500 and the sliding interval to 75 in this paper.

**Table 9:** Classification accuracy (%) under different sliding window lengths.

DataSet	(750, 75)	(625, 75)	(500, 75)	(375, 75)	(250, 75)
2a	78.54	82.68	**84.06**	82.13	81.52
2b	82.20	82.79	**85.09**	83.45	81.32

Boldface indicates the best performance.

#### 4.5.4 Different parameter selection for the model

The performance in terms of accuracy and parameter quantity of four model configurations (MC1, MC2, MC3, and MC4)on the 2a dataset is compared in Table 10. The results indicate that Configuration MC4 demonstrates the best overall performance. Although it has the smallest parameter count among the four configurations (only 22,000), it achieves an accuracy of 84.06%, which is only 0.20% lower than that of Configuration MC1 (84.26%), despite the fact that MC1 having a significantly larger parameter count of 188,000. In contrast, Configurations MC2 and MC3 both have 84,000 parameters and achieve the same accuracy of 82.35%. They neither offer an accuracy advantage nor require fewer parameters than Configuration MC4, thus making them suboptimal. Additionally, the comparison among Configurations MC2, MC3, and MC4 reveals that the model’s parameter count is primarily influenced by the number of $F_{1}$ and $F_{2}$ filters. Therefore, Configuration MC4 significantly reduces model complexity and computational cost while maintaining high accuracy, offering a clear efficiency advantage. This makes it particularly suitable for resource-constrained environments, such as the deployment on mobile or edge device. In summary, Configuration MC4 is the best-performing setup in this comparative experiment and represents the most suitable parameter combination for the current task requirements.

**Table 10:** Comparison of different network parameters of the model.

Index	Parameters	F1	F2	K1	K2	FC	Accuracy (%)
MC1	18.8w	24	48	128	8	96	84.26
MC2	8.4w	16	32	64	16	64	82.35
MC3	8.4w	16	32	64	32	64	82.35
MC4	**2.2w**	8	16	64	32	32	**84.06**

$F_{C}$ , mapping dimension. Boldface indicates the best performance.

## 5 Conclusion

A Data-Augmented Multi-Scale Temporal Attention Convolutional Network (AMANet) was proposed in this work to enhance motor imagery EEG classification performance. By augmenting limited samples, AMANet effectively mitigates overfitting caused by insufficient training data and adaptively capturing discriminative EEG features. To further assess the impact of architectural design, systematic analyses were conducted on convolutional kernel sizes, attention mechanisms, and sliding window lengths. Experimental results on the BCI Competition IV-2a,2b and high-gamma datasets demonstrate that AMANet achieves classification accuracies of 84.06, 85.09 and 95.48%, respectively, which significantly outperforms multiple benchmark methods and confirms its effectiveness in MI-EEG decoding. Ablation studies further reveal that both the multi-scale structure and the attention mechanism contribute positively to performance, and their integration enables more effective extraction of cross-temporal discriminative features. Although the proposed method has been validated against several classic and widely used baseline models, a limitation remains: we did not include some of the more recently proposed models characterized by substantially higher structural complexity and computational cost. Given that the primary goal of this work is to evaluate model suitability under small-sample conditions and limited computational resources, we deliberately selected comparison methods that are representative, stable, and practically reproducible. In future work we will expand the set of competing methods to include more complex state-of-the-art architectures for a systematic assessment of our approach across varying levels of model complexity. Furthermore, to enhance the practical utility of the model, we will focus on two key directions: first, improving cross-subject generalization to increase the model’s adaptability in real-world scenarios; and second, applying model quantization, parameter compression, and transfer-learning based techniques to reduce memory and computational footprint, thereby optimizing deploy ability in edge computing and other resource-constrained environments.

Where the number of EEG channels is denoted by C=22 The number of spatial filters in the CSP block is represented by $C_{1}$ , which is set to 12 for the 2a dataset and 2 for the 2b dataset. The number of sampling points is indicated by T =1,000 and $T_{1}$ =500. the number of temporal filters is represented by $F_{1}$ =8, and the number of pointwise filters is signified by $F_{2}$ =16. The sizes of different convolution kernels are denoted by $K_{1}$ =64 and $K_{2}$ =32 while the number of spatial filters is represented by D =2. SW indicates the sliding window, and *β* denotes the scaling range. Padding is applied during convolution to by which the spatial dimensions of the output tensor are preserved.

## Data Availability

Publicly available datasets were analyzed in this study. This data can be found here: https://github.com/robintibor/high-gamma-dataset.
